# Elementary Forms of Disease

**Published:** 1837

**Authors:** 


### X.—Elementary Forms of Disease.

*Illustrations of the Elementary Forms of Disease;* by Robert Carswell, M. D., Professor of Pathological Anatomy in the London University. Folio.—London, 1833, 4, 5, 6.

This splendid work was commenced in London, in 1833. It is published in separate numbers, each containing four colored plates, with appropriate references and descriptions. The plates are done on stone, from drawings executed by Professor Carswell; and when completed will form the most admirable series that has yet appeared on morbid anatomy. The following are the lesions represented in the ten numbers now before us: Tubercle, Carcinoma, Melanoma, Softening, Hemorrhage, Mortification, Pus, Hypertrophy, and Atrophy. It is not our design at present to do more than to express our favorable opinion of Dr. Carswell's work; on some future occasion we shall probably revert to it, and consider it in detail. How many more fasciculi are to appear, we are not able to say; but it is probable that there will be altogether as many as twenty-five or thirty. Those already published, cost about forty dollars; a sum which it is to be feared very few of our physicians will be able to devote to an object of this kind.

No individual in Great Britain is perhaps so well qualified for executing an enterprize of such magnitude and importance as Dr. Carswell. For many years a most zealous investigator of diseased structure in Paris, he was called soon after the organization of the University of London to the chair of Pathological Anatomy in that institution; a proof at once of his talents and attainments. Notwithstanding his arduous duties as a public teacher, Dr. Carswell has written more than almost any other English physician of his age. The London Cyclopædia of Practical Medicine contains a large number of highly valuable papers from his pen, nearly all the articles on morbid anatomy having been furnished by him; and the present work embraces more than one hundred folio pages of text and references. But more of Dr. Carswell and his beautiful work anon.

